# Childhood attention‐deficit hyperactivity disorder (ADHD) and cognitive function in later life: Analysis of HRS data

**DOI:** 10.1002/alz.091761

**Published:** 2025-01-09

**Authors:** Arne Stinchcombe, Douglas William Hanes

**Affiliations:** ^1^ University of Ottawa, Ottawa, ON Canada; ^2^ Bruyère Research Institute, Ottawa, ON Canada; ^3^ Renaissance School of Medicine at Stony Brook University, Stony Brook, NY USA; ^4^ Dalla Lana School of Public Health, University of Toronto, Toronto, ON Canada

## Abstract

**Background:**

Attention‐deficit hyperactivity disorder (ADHD) is a neurodevelopmental condition marked by cognitive deficits (e.g., challenges sustaining attention, distractibility). Symptoms of ADHD typically manifest in childhood and can interfere with engagement in school activities, chores, and interpersonal relationships. In many cases, symptoms endure into adulthood and older adulthood. ADHD in adulthood and mild cognitive impairment (MCI) share many overlapping cognitive (e.g., impaired executive functions) and non‐cognitive (e.g., depression, poor sleep) symptoms, complicating differential diagnosis. Currently, the association between ADHD and cognitive impairment associated with aging, such as MCI, remains unclear. The purpose of this present study was to examine differences in cognitive function by ADHD status in an aging sample.

**Method:**

Data were drawn from the Health and Retirement Study, a longitudinal survey of U.S. residents aged ≥51 years. Waves 4‐14 (1998‐2018) were used in the present analysis. Of the 10,804 participants included for analysis, 99 participants (i.e., 0.9% of the sample) reported having an ADHD diagnosis in childhood. Cognitive function was measured on a 27‐point scale. The primary analysis consisted of a series of multiple linear regression models, treating cognition as the outcome, and adjusting for age, sex/gender, wealth, education, and race.

**Result:**

Descriptive analysis showed that participants with ADHD reported lower levels of education and were more likely to have experienced homelessness and depression. Participants with ADHD showed lower cognitive scores at baseline (m = 14.52) compared to non‐ADHD peers (m = 17.01, t = 5.90, p<.001). Multivariable analysis revealed that after adjusting for covariates, ADHD status was associated with lower cognitive scores in the study (B = ‐1.88, p<.001). ADHD participants showed a similar slope of decline to non‐ADHD peers over time; however, cognitive scores were consistently lower at each time point for ADHD participants.

**Conclusion:**

Individuals with self‐reported childhood ADHD diagnoses demonstrated lower cognitive scores compared to their non‐ADHD counterparts, suggesting a persistent impact of ADHD on cognitive function in later life and potential risk for cognitive impairment. These findings emphasize the need for further research to elucidate the complex relationship between ADHD and cognitive impairment and underscore the importance of considering ADHD in the broader context of cognitive health throughout the lifespan.

